# Draft Genome Sequences of Two Listeria monocytogenes Strains Isolated from Raccoon Feces in Japan

**DOI:** 10.1128/mra.00495-22

**Published:** 2022-08-25

**Authors:** Shiori Yamamoto, Megumi Hasegawa, Eriko Iwabuchi, Hiroshi Asakura

**Affiliations:** a Division of Biomedical Food Research, National Institute of Health Sciences, Kawasaki, Kanagawa, Japan; b Department of Nutrition, School of Nursing and Nutrition, Tenshi College, Sapporo, Hokkaido, Japan; University of Maryland School of Medicine

## Abstract

Listeria monocytogenes serotype 4b strains RF01 and RF06 were isolated from raccoon feces in Japan. Here, we report the draft genome sequences of the two isolated strains; the genome sizes were 2,918,024 and 2,872,491 bp, with 535× and 510× coverage, for the RF01 and RF06 strains, respectively.

## ANNOUNCEMENT

Listeria monocytogenes is a causative foodborne pathogen of listeriosis and is widespread in the environment. This bacterium has also been isolated from raccoons (Procyon lotor), which are free-living animals; therefore, raccoons have been suspected to transmit L. monocytogenes by direct contact and/or indirect contamination of the environment via invasion of human residential areas (1, 2). In Japan, L. monocytogenes isolates from raccoons belong to serotype 4b, which is highly pathogenic for humans (3, 4). Furthermore, their pulsed-field gel electrophoresis (PFGE) patterns are highly similar to those of strains in humans (3). These strains may influence human health; however, the relevant information is limited. To characterize this genetic trait, in this study we sequenced L. monocytogenes isolates from raccoon feces.

Two strains of L. monocytogenes from raccoon feces, RF01 and RF06, were isolated in our previous study; samples collected from raccoons in cage traps were incubated in Fraser broth and *Listeria* Oxford medium (both from Becton Dickinson) at 35°C for 24 to 48 h (3). Genomic DNA was extracted from bacterial cultures that had been incubated on brain heart infusion agar at 37°C for 18 h using a Maxwell RSC blood DNA kit (Promega). After verification of the concentration and purity of DNA extracts using the TapeStation 4150 system (Agilent Technology), the genomic library was prepared using the NEBNext Fast DNA fragmentation and library prep set for Ion Torrent kit (New England Biolabs). Genome sequencing was performed using the Ion Chef/GeneStudio S5 system (Life Technologies). Raw reads were trimmed and assembled *de novo* using CLC Genomics Workbench v.21.0 (Qiagen) and annotated using the DDBJ Fast Annotation and Submission Tool (DFAST) v.1.1.4 (5). All analyses were conducted using the default parameters for the software programs unless otherwise specified.

In total, 7,061,058 and 6,820,342 raw reads (total lengths of 1,562,322,790 and 1,465,878,468 bp) were obtained from the RF01 and RF06 strains, respectively. These strains were assembled into 45 and 35 contigs, with accumulated lengths of 2,918,024 and 2,872,491 bp at 535× and 510× coverage, for RF01 and RF06, respectively (GC content, each 37.9%).

Their sequence types (STs) were identified as ST6 (RF01) and ST4 (RF06) via multilocus sequence typing (MLST) v.2.0 (6). L. monocytogenes ST6 has been reported to be a hypervirulent clone and is increasingly being associated with outbreaks (7); moreover, ST4 has been associated with meat products and is considered to highly contribute to listeriosis cases (8). The virulence factors of these strains were determined *in silico* using VirulenceFinder v.2.0. In total, 82 and 81 genes in RF01 and RF06, respectively, were identified (9). The data provide the genome sequence information for L. monocytogenes strains from raccoons and suggest that these strains carried by raccoons may potentially adversely affect human health. Further accumulation of data is needed for the epidemiological monitoring of the virulence potential of this pathogen.

### Data availability.

Draft genomes were deposited in DDBJ/ENA/GenBank under the accession numbers provided in Table 1. Raw sequence data were deposited in the DRA under the BioProject accession number PRJDB13507.

**TABLE 1:** Genome sequences of the isolated Listeria monocytogenes strains

Parameter	Data for strain:	
	RF01	RF06
Sequencing analysis		
No. of reads	7,061,058	6,820,342
Total read length (bp)	1,562,322,790	1,465,878,468
No. of contigs	45	35
Size (bp)	2,918,024	2,872,491
*N*_50_ (bp)	145,649	193,605
GC content (%)	37.9	37.9
Coverage depth (×)	535	510
No. of coding sequences	2,946	2,845
No. of rRNAs	3	4
No. of tRNAs	38	53
DDBJ accession no.	BRCB01000001 to BRCB01000045	BRCC01000001 to BRCC01000035
DRA accession no.	DRR362389	DRR362390
*In silico* analysis		
MLST ST	6	4
No. of virulence genes	82	81

## References

[1] Nowakiewicz A, Zięba P, Ziółkowska G, Gnat S, Muszyńska M, Tomczuk K, Majer Dziedzic B, Ulbrych Ł, Trościańczyk A. 2016. Free-living species of carnivorous mammals in Poland: red fox, beech marten, and raccoon as a potential reservoir of *Salmonella*, *Yersinia*, *Listeria* spp. and coagulase-positive *Staphylococcus*. PLoS One 11:e0155533. doi:10.1371/journal.pone.0155533. 27171434 PMC4865137

[2] Lee K, Iwata T, Nakadai A, Kato T, Hayama S, Taniguchi T, Hayashidani H. 2011. Prevalence of *Salmonella*, *Yersinia* and *Campylobacter* spp. in feral raccoons (*Procyon lotor*) and masked palm civets (*Paguma larvata*) in Japan. Zoonoses Public Health 58:424–431. doi:10.1111/j.1863-2378.2010.01384.x. 21824337 PMC7165867

[3] Hasegawa M, Iwabuchi E, Yamamoto S, Esaki H, Kobayashi K, Ito M, Hirai K. 2013. Prevalence and characteristics of *Listeria monocytogenes* in bovine colostrum in Japan. J Food Prot 76:248–255. doi:10.4315/0362-028X.JFP-12-278. 23433372

[4] Yoshida T, Takeuchi M, Sato M, Hirai K. 1999. Typing *Listeria monocytogenes* by random amplified polymorphic DNA (RAPD) fingerprinting. J Vet Med Sci 61:857–860. doi:10.1292/jvms.61.857. 10458115

[5] Tanizawa Y, Fujisawa T, Kaminuma E, Nakamura Y, Arita M. 2016. DFAST and DAGA: web-based integrated genome annotation tools and resources. Biosci Microbiota Food Health 35:173–184. doi:10.12938/bmfh.16-003. 27867804 PMC5107635

[6] Larsen MV, Cosentino S, Rasmussen S, Friis C, Hasman H, Marvig RL, Jelsbak L, Sicheritz-Pontén T, Ussery DW, Aarestrup FM, Lund O. 2012. Multilocus sequence typing of total-genome-sequenced bacteria. J Clin Microbiol 50:1355–1361. doi:10.1128/JCM.06094-11. 22238442 PMC3318499

[7] Nüesch-Inderbinen M, Bloemberg GV, Müller A, Stevens MJA, Cernela N, Kollöffel B, Stephan R. 2021. Listeriosis caused by persistence of *Listeria monocytogenes* serotype 4b sequence type 6 in cheese production environment. Emerg Infect Dis 27:284–288. doi:10.3201/eid2701.203266. 33350924 PMC7774546

[8] Henri C, Félix B, Guillier L, Leekitcharoenphon P, Michelon D, Mariet JF, Aarestrup FM, Mistou MY, Hendriksen RS, Roussel S. 2016. Population genetic structure of *Listeria monocytogenes* strains as determined by pulsed-field gel electrophoresis and multilocus sequence typing. Appl Environ Microbiol 82:5720–5728. doi:10.1128/AEM.00583-16. 27235443 PMC5007763

[9] Malberg Tetzschner AM, Johnson JR, Johnston BD, Lund O, Scheutz F. 2020. *In silico* genotyping of *Escherichia coli* isolates for extraintestinal virulence genes by use of whole-genome sequencing data. J Clin Microbiol 58:e01269-20. doi:10.1128/JCM.01269-20. 32669379 PMC7512150

